# Neighborhood Perception and Physical Activity in Later Life: Does Autonomy matter?

**DOI:** 10.1093/geroni/igaf122.2152

**Published:** 2025-12-31

**Authors:** SaeMi Park, W Quin Yow

**Affiliations:** Singapore University of Technology and Design, Singapore, Singapore; Singapore University of Technology and Design, Singapore, Singapore

## Abstract

This research examines the role of neighborhood perception in shaping physical activity engagement among Singaporean older adults, applying the Transactional Model of Stress and Coping. This study proposes a serial mediation model where neighborhood perception influences physical activity through perceived health and autonomy, with autonomy serving as a key coping resource. Using data from the 7th wave of the World Values Survey, this study analyzed a Singaporean society (N = 2007, Mage = 47.78, Range=21-91). PROCESS macro is used to test serial mediation as well as moderated mediation. Serial mediation analysis reveals that neighborhood perception indirectly influences physical activity through perceived health and autonomy (OR = 1.015, p = 0.012), though no direct effect of neighborhood perception on physical activity is observed. Furthermore, moderation analysis indicates that the moderating role of autonomy on perceived health is stronger among older adults (β=.018, p=.001), compared to younger adults, suggesting that autonomy is particularly important in later life to take advantage of the health benefits of favorable neighborhood environments. Findings highlight the cyclical linkage between perceived health and autonomy, which perpetuates and reproduces well-being in neighborhood among older adults. These findings confirm the erosion model within a stress and coping model, underscoring the deleting impacts of chronic exposure to stressor on psychological resources as well as contribute to the aging-in-place theory by highlighting the significance of autonomy in mitigating environmental stressors. Interventions fostering a sense of autonomy may enhance physical activity engagement among older adults, ultimately supporting healthier aging.

